# Movement ecology of an endangered mesopredator in a mining landscape

**DOI:** 10.1186/s40462-023-00439-5

**Published:** 2024-01-17

**Authors:** M. A. Cowan, J. A. Dunlop, L. A. Gibson, H. A. Moore, S. A. Setterfield, D. G. Nimmo

**Affiliations:** 1 Gulbali Institute, School of Agricultural, Environmental and Veterinary Sciences, Charles Sturt University, 386 Elizabeth Mitchell Drive, Thurgoona, NSW 2640 Australia; 2 School of Agriculture and Environment, The University of Western Australia, Crawley, WA 6009 Australia; 3 Department of Biodiversity, Conservation and Attractions, 17 Dick Perry Avenue, Kensington, WA 6151 Australia

**Keywords:** Northern quoll, Step-selection function, Habitat fragmentation, Accelerometer, Human–wildlife, Marsupial, Anthropogenic, Habitat selection, Foraging efficiency, Behaviour

## Abstract

**Background:**

Efficient movement and energy expenditure are vital for animal survival. Human disturbance can alter animal movement due to changes in resource availability and threats. Some animals can exploit anthropogenic disturbances for more efficient movement, while others face restricted or inefficient movement due to fragmentation of high-resource habitats, and risks associated with disturbed habitats. Mining, a major anthropogenic disturbance, removes natural habitats, introduces new landscape features, and alters resource distribution in the landscape. This study investigates the effect of mining on the movement of an endangered mesopredator, the northern quoll (*Dasyurus hallucatus*). Using GPS collars and accelerometers, we investigate their habitat selection and energy expenditure in an active mining landscape, to determine the effects of this disturbance on northern quolls.

**Methods:**

We fit northern quolls with GPS collars and accelerometers during breeding and non-breeding season at an active mine site in the Pilbara region of Western Australia. We investigated broad-scale movement by calculating the movement ranges of quolls using utilisation distributions at the 95% isopleth, and compared habitat types and environmental characteristics within observed movement ranges to the available landscape. We investigated fine-scale movement by quolls with integrated step selection functions, assessing the relative selection strength for each habitat covariate. Finally, we used piecewise structural equation modelling to analyse the influence of each habitat covariate on northern quoll energy expenditure.

**Results:**

At the broad scale, northern quolls predominantly used rugged, rocky habitats, and used mining habitats in proportion to their availability. However, at the fine scale, habitat use varied between breeding and non-breeding seasons. During the breeding season, quolls notably avoided mining habitats, whereas in the non-breeding season, they frequented mining habitats equally to rocky and riparian habitats, albeit at a higher energetic cost.

**Conclusion:**

Mining impacts northern quolls by fragmenting favoured rocky habitats, increasing energy expenditure, and potentially impacting breeding dispersal. While mining habitats might offer limited resource opportunities in the non-breeding season, conservation efforts during active mining, including the creation of movement corridors and progressive habitat restoration would likely be useful. However, prioritising the preservation of natural rocky and riparian habitats in mining landscapes is vital for northern quoll conservation.

**Supplementary Information:**

The online version contains supplementary material available at 10.1186/s40462-023-00439-5.

## Background

Movement and energy expenditure play a critical role in animal survival [1, 2]. Efficient movement, that is, the ability of animals to disperse and access resources (e.g., food, shelter) with minimal energetic cost, is a key factor determining their fitness and reproductive success [3, 4]. When resources are scarce, animals allocate more energy to obtain resources, which can reduce their fitness and increase mortality risk [5–7]. Hence, understanding the relationship between movement, energy use, and resource availability is important to inform conservation management [1, 8].

When anthropogenic disturbance is introduced to the landscape, it can influence animal movement [9–11]. Currently, between 75 and 95% of terrestrial land has been disturbed by humans globally [12–14]. The influence of disturbance on animal movement depends on how it alters the availability and distribution of threats and resources [15]. Animal movement efficiency can sometimes be improved in human-disturbed landscapes, due to the availability of anthropogenic food or shelter subsidies [16], reducing the distance required to access resources [17–20]. For example, grey wolves (*Canis lupus*) use linear human infrastructure like roads and seismic survey lines, to move faster and enhance their ability to search for prey [21, 22]. Other species may be negatively impacted by human disturbance, restricting movement by limiting access to natural habitat and reducing resource availability [9, 23, 24]. In some cases, this can even negatively increase animal movement, as the area required to gain sufficient resources is larger [5, 7, 10], requiring animals to move through low quality habitats to access areas where resources are high [25].

The spatial extent and configuration of low quality habitats can have flow on effects to movement [26]: moving through low quality habitats can reduce foraging efficiency while increasing mortality risk [27]. For example, the Amur tiger (*Panthera tigris altaica*), whose habitat has been severely fragmented by human activities, must cross busy roads and populated areas to reach favoured habitat patches, increasing their energy expenditure and risking human–wildlife conflict [28]. Disturbance can also introduce sublethal effects for animals, disrupting or altering other processes such as predator–prey interactions, genetic connectivity, and disease transmission [9, 29, 30]. Changes in movement patterns due to disturbance can also negatively affect fitness or breeding success, sometimes requiring more energy than in natural habitats to avoid increased threats or to search for scarce resources [10, 31, 32]. The presence of habitats which are attractive (e.g., offer shelter), but do not offer all required resources—or increase other threats to the animal—may lead to an ecological trap [33–35]. For example, African wild dogs (*Lycaon pictus*) can be attracted from protected areas into surrounding areas which are less safe, due to increased hunting success and lower competition with larger predators, but suffer higher mortality risks due to anthropogenic causes [36].

Mining is an anthropogenic disturbance responsible for large-scale habitat modification around the world [37, 38]. Globally, mining threatens more than 900 animal species [39], and threats to biodiversity from mining are expected to increase, with many mineral-rich regions also having high conservation value [40, 41]. Mining destroys and fragments natural habitats, introduces novel landscape elements such as pits, roads, large clearings, and waste rock dumps, and changes the distribution and abundance of resources such as shelter, water, and food [42]. With the increasing pressure of mining on natural habitats, it is important to understand how it affects animals and their movement and energy requirements [43–45].

**Fig. 1:**
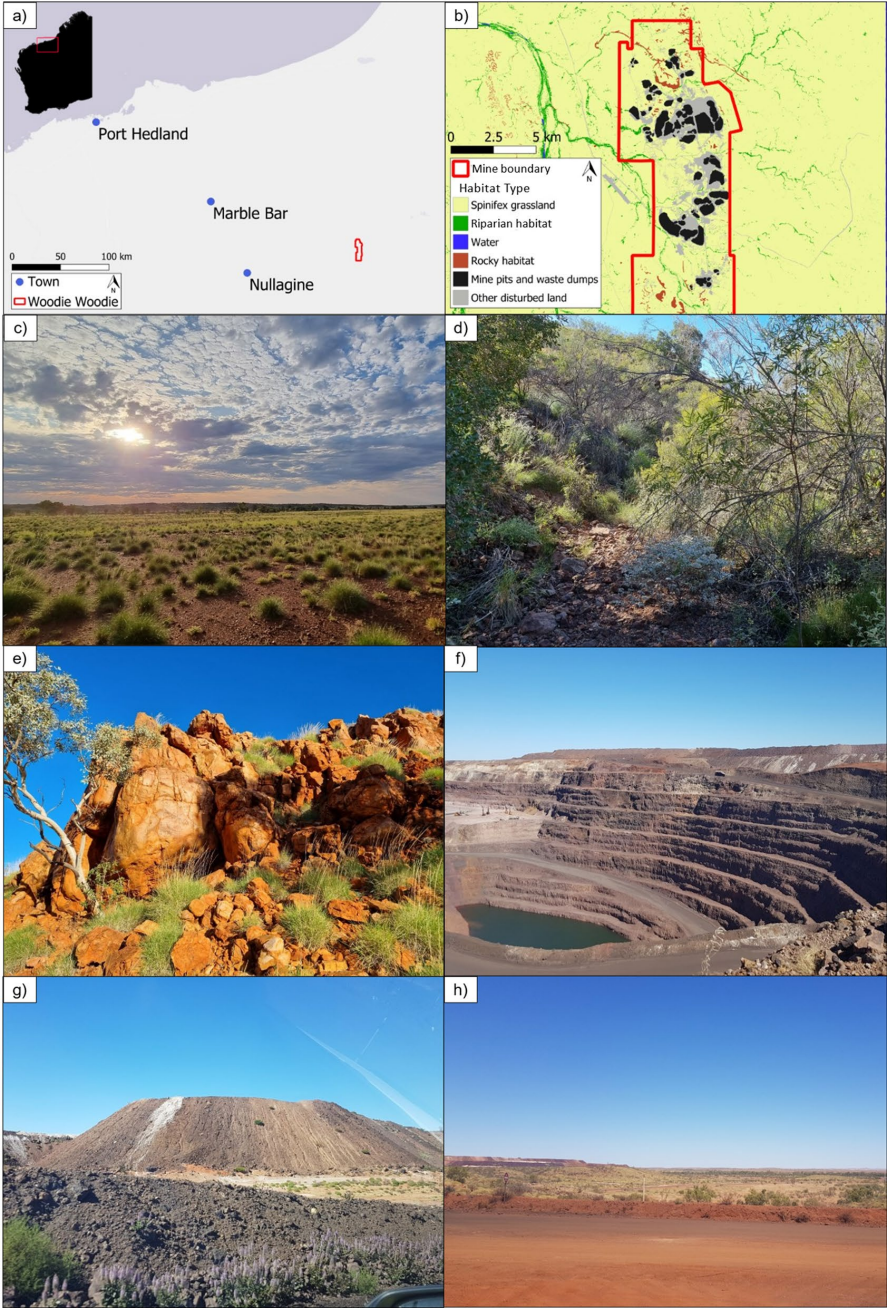
Maps of **a** the location of Woodie Woodie in the Pilbara landscape, and **b** habitat types at the site, including spinifex grassland, riparian habitat, water, rocky habitat, mine pits and waste dumps, and other disturbed land. The red line is the mine tenement boundary. Also included are photographs of the common habitats within the mining landscape: **c** spinifex grassland, **d** riparian habitat, **e** rocky habitat, **f** mine pits, **g** waste dumps, and **h** other disturbed land (e.g., roads, buildings, cleared areas)

We examined the effects of mining disturbance on the movement ecology and behaviour of an endangered marsupial mesopredator, the northern quoll (*Dasyurus hallucatus*)—*marlarlparra* in Nyamal language—in an extremely modified, active mining landscape. Drill and blast mining operations often target the rocky habitat that provides crucial denning sites for northern quolls [46–48], because of their rich deposits of minerals such as iron ore—removing complex rocky denning habitat and vegetation cover [49, 50]. While quolls do use structurally simple habitats like spinifex (*Triodia* spp.) grassland (Fig. 1) in natural landscapes—often when moving between patches of favoured rocky habitat [51]—they tend to be avoided where possible given the increased risk of encountering predators such as feral cats (*Felis catus*) and dingoes (*Canis lupus dingo*) [52, 53].

Mine sites are expected to be similarly unattractive to northern quolls as spinifex grassland, given the similar predator risk [54, 55], and presumed lack of denning resources compared to rocky habitat [48]. While there are some known instances of northern quolls interacting with human features such as rail infrastructure [56, 57] and rehabilitated habitats [47], our understanding of their movement and energy requirements in active mining landscapes remains poor [50]. We used GPS collars and accelerometers to investigate the broad and fine-scale habitat selection, and energy expenditure, of northern quolls living in an active mining landscape [58]. Based on existing knowledge of northern quoll movement and habitat use in natural landscapes, we predicted that:

1. At the broad scale, northern quolls will use areas with lower proportions of disturbed mining habitat and higher proportions of rugged, rocky habitat than what is available in the landscape, likely due to a lack of resources (e.g., dens, vegetation) in mining compared to rocky habitats.
2. At the fine scale, quolls will avoid mining habitats (e.g., cleared areas, roads) and select for rocky habitats and areas close to rocky habitat, due to an expected higher risk of predation and lack of key resources (e.g., dens, food) in mining compared to rocky habitats.
3. When quolls do traverse mining disturbed habitats in the landscape, the energetic costs will be higher than in natural habitats—where food and den availability is likely to be higher—because it will take longer to gain resources.

We use our results to provide management recommendations for the conservation of northern quolls in areas of active mining and make suggestions about the use of disturbed landscapes by small-medium sized mammals.

## Methods

### Study species

The northern quoll is a small-medium sized (~ 300–600 g) marsupial mesopredator native to northern Australia [59]. The northern quoll is an oppurtunistic omnivore, eating a range of vertebrate, invertebrate, and flora species [59]. Over the past century, northern quolls have suffered substantial range declines, largely due to a combination of habitat loss, introduced predators, altered fire regimes, and invasive cane toads (*Rhinella marina*)—a toxic species of toad that is lethal to quolls when consumed [60, 61]. Except for the cane toad, all these threats are present in the Pilbara region of Western Australia, yet there has been relatively little range contraction here compared to other regions of northern Australia [60]. Nonetheless, northern quolls are listed as Endangered under IUCN, national EPBC, and Western Australian state listings [62, 63].

### Study area

This study was undertaken is the Pilbara bioregion of northern Western Australia. The region experiences a semi-arid climate, with average maximum temperatures of 37°C during summer and 25°C during winter [64]. Annual rainfall is variable (250–500 mm), with most falling between December and February [65]. The region is characterized by ancient, topographically rugged rocky terrain, deep gorges, and rough escarpments, interspersed with expansive hummock grasslands [66, 67]. The grasslands are dominated by a *Triodia* spp. (spinifex) ground layer (to < 1 m), with a sparse *Acacia* and *Eucalyptus* spp. upper story. Plant species composition is largely influenced by the local fire history and geology. Rocky habitat contains a mixture of grass, *Eucalyptus*, and *Ficus* species littered amongst embedded and scattered rock [67, 68]. Drainage lines (e.g., creeks) typically have a narrow riparian zone, with a dense layer of grass species, shrubs, and trees (e.g., *Acacia* and *Eucalyptus* spp.), while they are often associated with rocky features [67].

Quolls were monitored at the Woodie Woodie mine, located on Nyamal Country in the eastern Pilbara region, bordering the Great Sandy Desert and close to the eastern edge of the northern quoll’s Pilbara range [60; Fig. 1]. Woodie Woodie is an active manganese mine managed by Consolidated Minerals (ConsMin), which has been in operation since the early 1950’s and has historic and active mining disturbance. Active mining occurs 24/7 with night operations aided by spotlights. The mine site is situated within Warrawagine station and cattle grazing occurs on and around the mine. Woodie Woodie employs an open pit mining strategy and is surrounded by natural habitat encompassing spinifex grassland, riparian habitat, and rocky habitat (Fig. 1). The mine site is made up of open pits, waste rock dumps (henceforth waste dumps), buildings, roads, flat cleared areas, and remnant patches of natural habitat. All areas of disturbed and undisturbed habitats, including mine pits and waste rock dumps are accessible to northern quolls. The mining footprint is approximately 127 km^2^ (12,700 ha). The Oakover River runs south to north ~ 6 km east of the mine with many non-perennial tributaries feeding this river from around and through the mine site (Fig. 1). The mine site also contains patches of permanent water within historic mine pits, storage ponds, and pools in sections of drainage lines. All data included in this study were collected from individuals caught within the mine tenement boundary (Fig. 1).

### Data collection

We completed two data collection periods at Woodie Woodie, one during part of the northern quoll breeding season when females begin to have pouch young (September–October 2021) and another during part of the non-breeding season the following year, before mating began (June–July 2022) [69]. Northern quolls were trapped using wire cage traps (45 cm × 17 cm × 17 cm, Sheffield Wire Co., Welshpool, WA). We set a maximum of 50 cage traps per night, in different parts of the mine, targeting areas where northern quolls had been sighted previously or areas that were likely to contain northern quolls based on the availability of den sites and food (e.g., native *Ficus* spp.). Initial trapping was undertaken for 14 nights in each season. Traps were moved around the mine tenement to avoid capturing the same individuals and to ensure we captured quolls from a range of areas and habitats. Traps were placed in transects ~ 50–100 m apart in rocky and riparian habitats where northern quolls den [48]. These areas were often close to mining disturbance (Fig. 1). Traps were baited with a mixture of oats, peanut butter, and sardines [as per 70]. Traps were opened in the evening and checked and closed the following morning.

All individuals were processed at the site of trapping. A series of morphological measurements were recorded, including sex, weight, testes size (for males), hindfoot length, and tail base circumference. Animal ethics permits required collar weight to not exceed 5% of the animal’s body weight, so we attached a 20 g LiteTrack 20 RF GPS Collar (Lotek, Havelock North) to animals weighing 400 g and above. Twelve GPS collars were deployed for ~ 30 days, although all stopped recording data before retrieval due to memory and battery limitations (mean ± SE; 20 ± 1.5 nights after cleaning). One male individual was collared in breeding and non-breeding season. To retrieve GPS collars, we set targeted traps around daytime den locations, located using very-high frequency (VHF) transmitters inside each collar. Upon successful overnight trapping, the GPS collar was removed the following morning, and a general health check was undertaken in the field, including repeating the morphological measurements taken in the initial trapping. The quoll was then released.

GPS collars were set to record GPS locations between 6 pm and 6 am to target the time when northern quolls are most active [47, 71], and to avoid unsuccessful fixes draining the battery while quolls were denning. GPS collars were set to record locations every 30 min, resulting in a possible 24 fixes per night. This fix rate enabled frequent recordings to investigate fine-scale habitat use, but also enabled a longer monitoring period given battery limitations with a small GPS tracker. GPS collars also contained accelerometers and temperature loggers. Accelerometers were set to record raw acceleration (*g*) and temperature (°C) at 5 s intervals, 24 h a day. Accelerometers measured acceleration at a range of ± 4G and temperature monitoring was accurate to ± 0.5 °C. Raw acceleration was recorded on 3 axes—X, Y, and Z—representing surge (forward-backwards movement), heave (upwards-downwards movement), and sway (sideways movement). Accelerometers were positioned at the bottom of the collars, sitting under the chin of the quoll. However, because collars were circular, we could not guarantee that they would always remain in perfect orientation due to the collars’ rotational ability. We accounted for this during analyses (see below).

To quantify northern quoll movement in relation to landscape variables, we used QGIS v3.12 [72] and RStudio version 2022.07.2 [73] to create four maps of landscape features: (1) habitat type, (2) topographic ruggedness index (TRI), (3) distance from disturbance, and (4) distance from rocky habitat. Habitat type, distance from disturbance, and distance from rocky habitat were mapped at a 10 m scale, while topographic ruggedness index was mapped at a 12.5 m scale. Habitat types on the habitat map included spinifex grassland, riparian habitat (dense vegetation associated with drainage lines), rocky habitat, and mining disturbed land. During breeding season, quolls were sometimes tracked to dens within rocky waste dumps and mine pits, therefore, we split mining disturbed land into two types: (1) mine pits and waste dumps, and (2) other disturbed land (e.g., roads, buildings, and large cleared areas). To compare the environmental characteristics of each habitat type, we extracted the mean NDVI values (10 m scale) and the median topographic ruggedness for each habitat type from the *observed* and *available* landscapes. For detailed methods of map creation and layer details, see Additional file 1: Appendix 1.

### Data processing and preparation

To eliminate GPS errors, we cleaned and processed all data before analyses. All data processing and analyses were performed in RStudio version 2022.07.2, unless stated otherwise [73]. We removed all GPS points with a horizontal dilution of precision (HDOP) > 10, due to the decreased accuracy of these GPS points [74–76]. This resulted in the removal of 59 GPS points. Fix errors often increase with increasing HDOP, but the suitability of using HDOP as a filtering method is debated [77]. Some fixes with a high HDOP can also have low error, meaning that the removal of high HDOP fixes can remove some accurate locations [78, 79]. However, the effective use of a HDOP limit can remove major outliers from data—important for fine scale movement analysis—while limiting data loss [74, 80], with only a 3% data reduction seen here. Four trial GPS collars in fixed locations had a mean ± SE locational error of 8.3 ± 0.46 m when HDOP was < 10. After this, any remaining unrealistic GPS fixes were removed based on the average maximum speed of northern quolls (4.5 m s^−1^) [81], where points too far to be reached in the time between fixes were excluded [82]. This resulted in one location being excluded. We screened data further by discarding all GPS points on the night prior (6 pm–12 am) and morning of (12 am–6 am) any captures, to avoid locations when an individual was in a trap. One individual was discarded from all analyses, as fix success was extremely low and there were too few fixes to undertake movement analyses [24, 83, 84]. For one collared individual which was predated upon by a feral cat after 20 nights (confirmed by DNA analysis), we removed all GPS points from 6 pm onwards on the night of the predation event (time of event ~ 7:30 pm).

To investigate the habitat selection of northern quolls, we converted all GPS points to movement steps (henceforth ‘observed steps’) using the R package ‘amt’ [85]. To ensure movement was standardised by time, we removed all locations that were separated by more than 30 min, as longer time periods would likely miss finer movements between GPS fixes [86]. This resulted in the creation of several bursts (isolated groups of steps with a 30-min sampling rate). We removed all bursts with less than three movement locations, the minimum required to calculate turning angles [85]. Further data processing is outlined in the Data Analysis—Fine Scale Habitat Selection section when describing the fitting of integrated step selection functions (iSSFs).

Raw acceleration data (*g*) measured by accelerometers were converted from raw acceleration ($X,$ $Y,$ $Z$) to the vector of the dynamic body acceleration (VeDBA)—which has been used for northern quolls previously [71]. VeDBA is adept as a proxy for energy expenditure and deals with variation in accelerometer orientation (caused by potentially rotating collars) better than other measures such as overall dynamic body acceleration (ODBA) [87, 88]. Raw accelerometer data were first converted to dynamic body acceleration (DBA) by smoothing each axis to obtain the static acceleration using a running mean over 10 s (encompassing two consecutive values), then subtracting the static acceleration from the raw values for each 5 s measurement [87]. The dynamic body acceleration values were then converted to positive values, and we calculated VeDBA using:

$$VeDBA \, = \sqrt {A_{X}^{2} + A_{Y}^{2} + A_{Z}^{2} }$$

where $A$ is the dynamic body acceleration corresponding to the $X,$ $Y,$ and $Z$ axes of the accelerometer [87]. We then calculated the mean VeDBA in each 30-min *observed* movement step for each individual quoll. This meant that all mean VeDBA values recorded between 6 am and 6 pm when GPS collars were not recording, were discarded for this analysis.

Only one female was captured that was large enough to be collared, therefore all individuals were pooled regardless of sex. In non-breeding season, when the female quoll was collared, home ranges are relatively similar between sexes and habitat selection is not likely to differ substantially [59, 89].

### Data analysis—broad scale habitat selection

To identify the broad scale habitat selection of northern quolls, we estimated northern quoll movement ranges. Movement ranges represent the broad-scale space which animals use and encompass all habitats that they may access during movement [90]. GPS tracking time was not long enough for quolls to cover their complete home range; thus, none reached an asymptote when fixes were added sequentially (i.e., over time) at 10-fix intervals [91]. We therefore refer to the broad areas used by quolls during tracking as ‘movement ranges’, which were estimated from utilisation distributions (UDs) at the 95% isopleth using fixed kernel density estimation (KDE) [92, 93]. The UD represents a probabilistic density function that estimates how frequently an animal is expected to be found in a particular area [94]. The 95% isopleth refers to the area where the animal has a 95% probability of being found, excluding potential outliers [95]. We used the ad hoc method *(h*_*ad hoc*_) to determine *h*—which is the bandwidth (smoothing) parameter that dictates the width of the kernel function used to estimate the probability density function [96]—because *h*_*ad hoc*_ is robust to sample size, accurately reflects the observed movement range, and is consistent and repeatable [97, 98; Additional file 1: Table S1]. We used the package “rhr” in R to estimate movement ranges [99].

To compare the composition of landscapes within ‘*observed’* quoll movement ranges with what was available in the landscape, we identified the area of the landscape considered accessible but not visited by an individual [100]. To define the boundaries for the available landscape for each quoll, we followed methods described in Cowan, Moore [97] and Wysong, Hradsky [101]. We fit a 100% minimum convex polygon (MCP) to the GPS data of each quoll. We then placed a buffer around each MCP equal to the radius of the largest *observed* movement range (5220 m) and subtracted the radius of the activity area being measured [97]. Within the available area, we randomly generated five circular ‘*available*’ movement ranges per individual, which were equal in size to the movement range of the individual being measured [97, 101, 102]. We overlaid the *observed* (*n* = 9) and *available* (*n* = 45) movement ranges on habitat maps (see Additional file 1: Appendix 1), then used the ‘extract’ function in the “raster” package to determine the proportion cover of each habitat type (spinifex grassland, riparian habitat, rocky habitat, mine pits and waste dumps, other disturbed land), as well as the median topographic ruggedness, mean distance from disturbance, and mean distance from rocky habitat for each *observed* and *available* movement range [103].

Next, to determine if northern quolls preferred to locate their *observed* movement ranges in areas with certain environmental characteristics compared to what was *available*, we fit zero-inflated beta regression models for proportion data and we fit generalised linear mixed regression models (GLMMs) with a Gaussian distribution for continuous data in R. We did not have enough data to accurately model seasons separately, so data were combined for breeding and non-breeding season. For zero-inflated beta regression models to determine selection for different habitat types, we fit separate models for each habitat type, where the response variable was the proportion of that habitat within the movement range, and the predictor variable was the movement range type (*observed* or *available*) [104]. We allowed the zero-inflation component to vary for each movement range type, which models the excess zeros in the response variable—that can occur when the habitat type is not present for some observations and helps to deal with overdispersion [104]. We included a random effect of individual to account for the non-independence of multiple *available* movement ranges for the same individual, and to capture the variability in the intercepts across different individuals [105]. Second, to determine if northern quolls selected movement ranges with differing topographic ruggedness, distance from disturbance, or distance from rocky habitat compared to the available landscape, we fit GLMMs where the response variable was the environmental variable (i.e., median topographic ruggedness, mean distance from disturbance, or mean distance from rocky habitat) and the predictor variable was the movement range type (*observed* or *available*) [106]. We classified a Gaussian distribution as data were continuous and again included a random effect of individual [105, 107]. Significant differences between *observed* and *available* movement ranges were identified if 95% confidence intervals did not cross zero. Zero-inflated beta regression models and GLMMs were fit using the ‘brm’ function from the “brms” package in R [106].

### Data analysis—fine scale habitat selection

To investigate how northern quolls interact with mining disturbance, as well as with other environmental variables at the fine scale, we used integrated step selection functions (iSSFs) [8]. Step selection functions involve a form of conditional logistic regression where each ‘*observed* step’ (path connecting two consecutive observed locations of the individual) is compared with a set of ‘*available* steps’ with a strata term (step ID) which pairs *observed* steps with their respective *available* steps [86, 108, 109]. Integrated step selection functions take this further, by including animal movement and resource selection parameters in the model, reducing bias, and allowing further estimation and simulation of habitat selection [85, 110, 111]. For each *observed* step, we generated five random *available* steps with turning angles drawn from a von Mises distribution and step lengths drawn from a gamma distribution [8, 86, 112, 113]. For each *observed* and *random* step, we extracted covariates from the four landscape maps at the end of the step. We constructed separate models for the breeding and non-breeding seasons due to differences in northern quoll behaviour during these periods [89].

When including multiple individuals in an iSSF model, it is common to account for individual variation in selection [105]. However, individual-specific random slopes are extremely difficult to fit in conditional logistic regression [86, 105, 114]. There are other options, including mixed effects modelling of step-selection functions [105], however, we did not have enough strata per individual to fit these [115]. Therefore, to allow inference of population effects and further analysis of relative selection strength (RSS), we assumed homogeneity across individuals and pooled data by season for iSSF analyses [85, 115]. We fit iSSFs using the ‘fit_issf’ function in the “amt” package in R [85].

For our global model, the response variable was the case (i.e., an *observed* or *random* step) [85, 108]. Predictor variables included the four landscape variables extracted from the end of each *observed* and *random* quoll step (i.e., the habitat type, topographic ruggedness, distance from disturbance, and distance from rocky habitat). Rocky habitat was set as the reference category (i.e., the intercept) for habitat type. Habitat selection depends on the scale at which resources are distributed throughout the landscape and how animals move between them, so we included the log_10_ of the step length and the cosine of the turning angle as covariates in iSSF analyses [110]. Unexpectedly, there was a high survival rate of males living into their second year (two collared males in non-breeding season). Male northern quolls often die off after their first breeding season [116]—therefore, for quolls tracked during the non-breeding season, we included an interaction of age with the log_10_ of the step length and the cosine of the turning angle to determine if second year quolls moved differently to first year individuals. We included ‘step ID’ as a strata term in both models to ensure *observed* steps were paired with their respective *available* steps [85]. We checked categorical and continuous variables for correlation using Pearson’s *r*, ANOVA, and chi-square tests. Water was excluded from analyses due to a lack of representation.

We compared the global model with simplified variations of the model separately for each season [115; see Additional file 1: Table S2, for simplified model structures, 117, 118]. We undertook model selection using Akaike’s Information Criterion corrected for small sample bias (AICc) using the ‘aictabCustom’ function in the “AICcmodavg” package in R [119]. Models were regarded as having substantial support when ΔAICc < 2 [120, 121].

The global model had substantial support in both seasons (see Results). Therefore, we undertook relative selection strength (RSS) analysis using this model, so we could investigate the potential influence of all environmental variables on northern quoll fine-scale habitat selection [85, 115, 122]. For categorical covariates, the RSS estimates the probability of an animal selecting a particular habitat relative to another, and for continuous covariates, it measures the probability of selection across differing values, while holding all other covariates constant [122]. This approach is appropriate for observed-available designs such as this [115, 122]. To determine northern quoll RSS for each covariate, we first updated the selection-free movement kernel with the habitat selection coefficients of the global models to be able to estimate the RSS using both the movement (e.g., the log_10_ of the step length) and habitat selection parameters—which reduces bias [85, 114]. This was done separately for each season. RSS was calculated using the ‘log_rss’ function in the “amt” package [85]. We present the RSS for all habitat types, distance from disturbance, and distance from rocky habitat, and present the log(RSS) for topographical ruggedness to better visualise the relationships between seasons.

### Data analysis—energy expenditure

To examine the influence of mining disturbance and other landscape features on mean VeDBA (i.e., energy expenditure), we combined spatial and accelerometry data [123–125], using a piecewise structural equation modelling (PSEM) approach. We did this with the ‘psem’ function from the “piecewiseSEM” package in R [126]. PSEMs are a statistical approach used to analyse multiple complex interacting variables by uniting them into a single model [126]. They are useful for investigating direct and indirect effects of multiple predictor variables on response variables, and for examining causal relationships in ecological systems [126, 127]. Unlike classical SEMs, where global estimation is used to construct a model, the piecewise approach allows each response variable to be modelled separately as simultaneous generalised linear mixed-effects models (GLMMs) [127, 128]. We fit a separate PSEM for each environmental variable, which were; the proportions of each habitat type, the median topographic ruggedness index (TRI), the mean distance from disturbance, and the mean distance from rocky habitat, for each *observed* step [86, 108]. The first GLMM in each PSEM included step length as the response variable and the environmental variable as well as mean temperature (C°) as predictor variables (mean temperature was controlled for in all PSEMs). The second GLMM within each PSEM included mean VeDBA as the response variable and the environmental variable, mean temperature, and step length as predictor variables. Both models included a random effect of individual to account for repeated measurements by the same individual [129]. We calculated the conditional *R*^*2*^ for each GLMM to determine the variance explained by the predictor variables [128, 130]. For each GLMM within the PSEM, we calculated the relevant range coefficients—which represent the standardised effects of the predictor variables on the response variables for each model, and are useful for comparing the relative effects of predictors [126, 131]. We also calculated the total effect of each environmental variable on mean VeDBA both directly and indirectly as mediated through step length. This was done by calculating the partial correlation between the environmental variable and mean VeDBA while controlling for step length (direct effect), calculating the partial correlation between step length and mean VeDBA while controlling for the environmental variable (indirect effect), and then summing both the direct and indirect effects [132]. Finally, we fit a single PSEM with the inclusion of season as a predictor variable, to test its effect on mean temperature, step length, and mean VeDBA.

## Results

In total, we tracked 12 northern quolls across two seasons at Woodie Woodie. Of these individuals, two collars were not recovered. Of the remaining 10 individuals, one was excluded from analysis due to a lack of data. This left us with nine individuals for analysis: four from the breeding season and five from the non-breeding season (Additional file 1: Figure S1; Figure S2; Figure S3; Figure S4). Only one female was collared (in non-breeding season; Table 1), and one collared male was predated by a feral cat on a waste rock dump in the mining landscape. Individuals were smaller and had decreased tail circumference in breeding season compared to non-breeding season, whereas foot length was similar, and testes were larger in breeding season (Table 1). Following data cleaning, the average number of days with GPS locations for the nine individuals was 20.50 ± 0.50 days in breeding season, and 21.20 ± 2.97 days in non-breeding season. The average number of total fixes after data cleaning was 257 ± 10 fixes for breeding season, while fix success was lower in non-breeding season, with an average of 186 ± 49 fixes per individual (Table 1).

**Table 1:** The body condition and tracking details of the nine northern quolls used in analyses

Season	ID	Sex	Age (years)	Fixes	Days	Weight (g)	Foot length (mm)	Tail circ. (mm)	Testes diameter (mm)	Movement range (ha)
Breeding	33421	M	1	274	22	640	34.80	45	23.20	6584.67
	33427	M	1	260	20	540	37.90	55	24.50	6331.69
	33411	M	1	227	20	545	36.60	42	21.20	3115.55
	33425	M	1	266	20	640	38.20	50	24	8576.21
	Average		1	257	20.50	591	36.90	48	23.20	6152.03
	SE		0	10	0.50	28.16	0.77	2.86	0.73	66.79
Non-breeding	33415	F	2	99	17	450	31.30	60	NA	157.87
	33413	M	1	92	13	855	37.50	69	16.30	1169.05
	33423	M	2	226	25	805	34.30	65	14.90	723.78
	33412	M	1	356	30	760	38.20	71	20.10	85.99
	33422	M	2	158	21	700	35.20	55	21.60	387.46
	Average		1.6	186	21.20	714	35.30	64	18.20	504.83
	SE		0.24	49	2.97	70.77	1.23	2.93	1.57	199.84

**Fig. 2:**
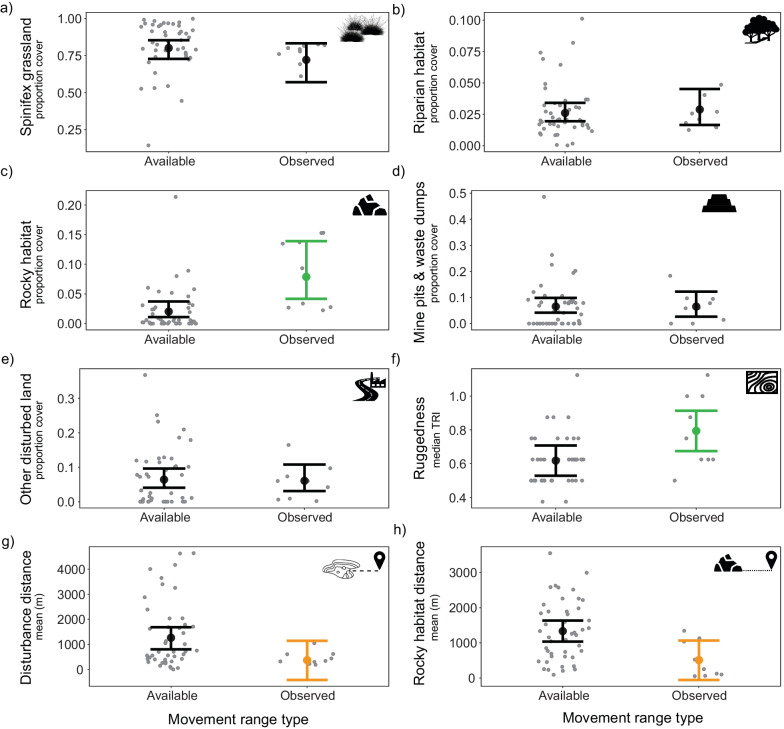
Conditional effects plots for all models analysing the influence on broad scale northern quoll movement ranges by the proportional cover of **a** spinifex grassland, **b** riparian habitat, **c** rocky habitat, **d** mine pits and waste dumps, and **e** other disturbed land, as well as the **f** median topographic ruggedness index, **g** mean distance from disturbance, and **h** mean distance from rocky habitat. Small grey points indicate raw data, large points indicate the conditional effect for each model, and bars indicate the 95% CIs. Green bars indicate a significant positive relationship for *observed* compared to *available* movement ranges, orange bars indicate a significant negative relationship for *observed* compared to *available* movement ranges, and black bars indicate a non-significant relationship

On average, northern quolls had movement ranges which were more than 12 times larger in breeding season compared to non-breeding season (Table 1; Additional file 1: Figure S1; Figure S2). Northern quoll observed movement ranges had a higher proportion cover of rocky habitat compared to the available landscape, and all other habitats were used in proportion to their availability (Fig. 2; Additional file 1: Table S3). Northern quoll movement ranges were in the most topographically rugged areas of the landscape, and were, on average, closer to rocky habitat and disturbed mining habitats relative to the available landscape (Fig. 2; Additional file 1: Table S3).

**Fig. 3:**
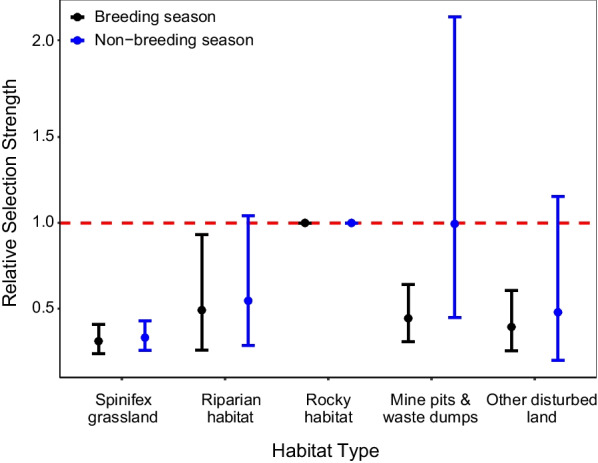
The relative selection strength (RSS) for northern quolls in breeding and non-breeding season, where the selection of spinifex grassland, riparian habitat, mine pits and waste dumps, and other disturbed land is compared relative to selection for rocky habitat. Points reflect the RSS and bars reflect the 95% CIs. Black points and bars reflect selection in breeding season while blue points and bars reflect selection in non-breeding season. The red dashed line reflects the relative selection strength for rocky habitat and a significant difference is observed if CIs do not cross this line

**Fig. 4:**
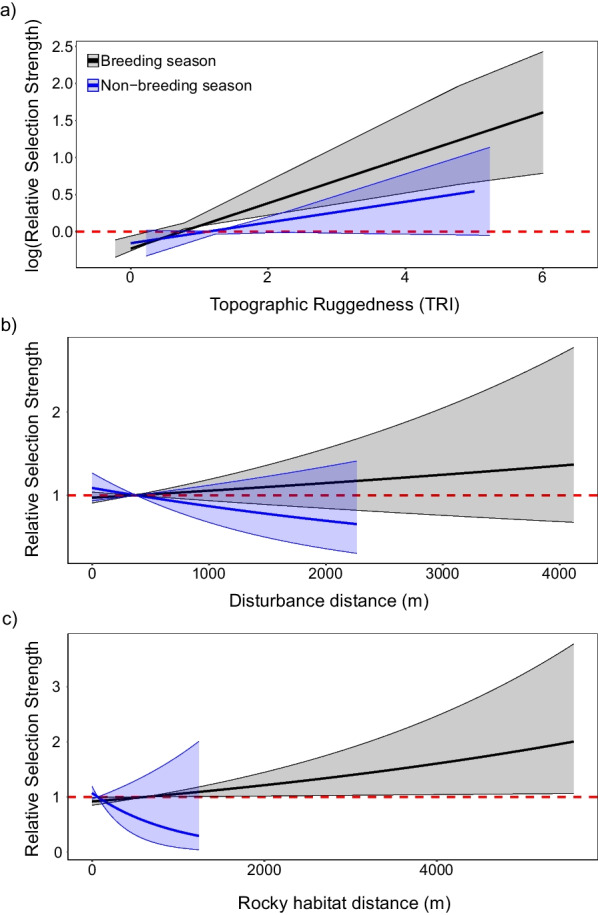
The **a** log relative selection strength (logRSS) by northern quolls for topographic ruggedness, as well as the RSS for distance from **b** disturbance and **c** rocky habitat. The black line reflects breeding season while the blue line reflects non-breeding season. Shaded areas reflect the 95% confidence intervals. The red dashed line reflects the median topographic ruggedness, the mean distance from disturbance, and the mean distance from rocky habitat respectively. Selection above or below this line reflects the relative selection strength being higher or lower than the median or mean

In terms of fine-scale habitat selection, which refers to active night-time movements, the global iSSF model had substantial support in both seasons (Additional file 1: Table S2). The global iSSF model for breeding season suggests that, within their movement range, northern quolls were significantly less likely to use mining habitats, spinifex grassland, and riparian habitat compared to rocky habitat. However, the global iSSF model for non-breeding season suggests that, within their movement range, northern quolls used both mining habitats and riparian habitat at a similar rate to rocky habitat, but used spinifex grassland significantly less (Fig. 3; Additional file 1: Table S4). Northern quolls selected for areas with higher topographic ruggedness relative to the landscape median in breeding season but did not show significant selection preferences for topographic ruggedness in non-breeding season (Fig. 4). Distance from disturbance had no effect on northern quoll fine scale habitat selection during either season, while quolls selected for areas which were further from rocky habitat relative to the landscape mean in breeding season, with no selection preference in non-breeding season (Fig. 4). Age had no influence on northern quoll step length or turning angle (Additional file 1: Table S4). When undertaking VHF tracking of northern quolls during the day, we observed northern quolls denning within a mixture of rocky habitat, mine pits, and waste dumps.

Among all habitat types, riparian habitat had the highest mean NDVI value ± SD (0.36 ± 0.11), followed by rocky habitat (0.19 ± 0.05), spinifex grassland (0.16 ± 0.04), other disturbed land (0.11 ± 0.04), and mine pits and waste dumps (0.09 ± 0.04; Additional file 1: Table S5). Rocky habitat had the highest median topographic ruggedness ± IQR (1.25 ± 1), followed by spinifex grassland, mine pits and waste dumps, and other disturbed land which all had a median TRI of 0.5 ± 0.375. Riparian habitat had the lowest median topographic ruggedness (0.375 ± 0.375). Mine pits and waste dumps had the highest maximum topographic ruggedness (6.875).

**Fig. 5:**
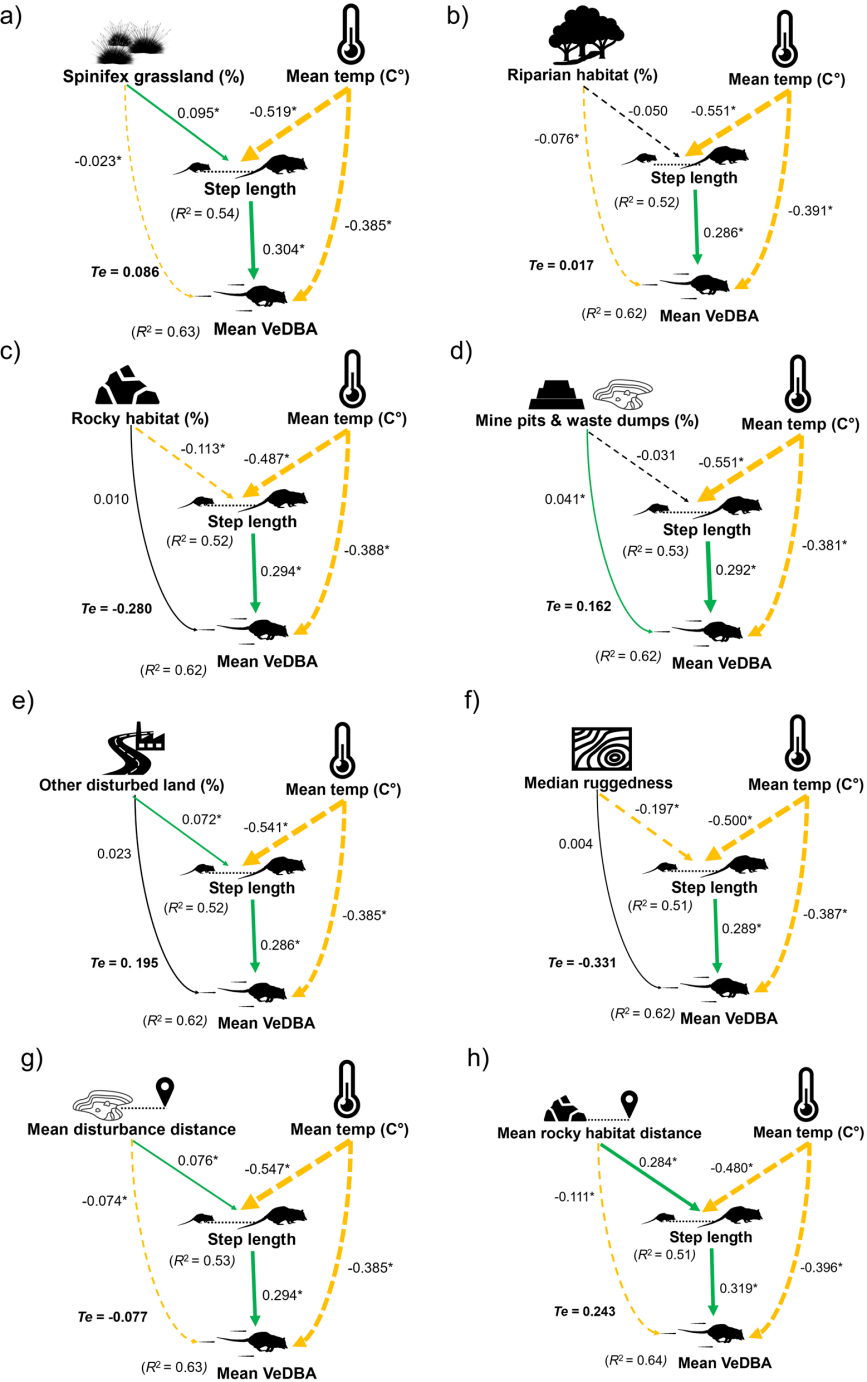
Relevant range coefficients for step length and mean VeDBA (a proxy for energy expenditure) related to the influence of mean temperature and the proportion used of **a** spinifex grassland, **b** riparian habitat, **c** rocky habitat, **d** mine pits and waste dumps, **e** other disturbed land, as well as **f** median ruggedness, **g** mean distance from disturbed land, and **h** mean distance from rocky habitat. Dashed arrows represent a negative relationship and solid arrows represent a positive relationship. Arrow colour represents significance (*p* =  < 0.05) with green representing significant positive relationships, orange representing significant negative relationships, and black representing a non-significant relationship. Arrow width reflects the size of the effect, with wider arrows representing a larger effect. An asterisk on relevant range coefficients also signifies that the relationship is significant and the conditional *R*^2^ value for step length and mean VeDBA is listed for each model, outlining the variance explained by the predictor variables. *Te* represents the total effect coefficient of each environmental variable on mean VeDBA, both directly and mediated through step length

PSEMs demonstrated that using higher proportions of spinifex grassland increased northern quoll step length, with a total effect of 0.086 on mean VeDBA, effectively raising mean VeDBA by 8.6% per unit increase despite its negative direct effect (Fig. 5). Riparian habitat had a similar, though less pronounced total effect, with an increase of 1.7%. Conversely, higher proportions of rocky habitat led to shorter step lengths, decreasing mean VeDBA by 28% per unit increase (Fig. 5). Mine pits and waste dumps had the highest positive total effect on mean VeDBA raising VeDBA by 16.2% per unit increase. Using higher proportions of other disturbed land also resulted in longer step lengths, contributing to a 19.5% increase in mean VeDBA per unit increase (Fig. 5).

Median ruggedness, similar to rocky habitat, had a negative total effect on VeDBA, mainly due to shorter step lengths with increased ruggedness, resulting in a 33.1% decrease in mean VeDBA with every unit increase in median ruggedness (Fig. 5). Increasing distance from mining disturbance also had a negative total effect on mean VeDBA (7.7%), whereas increasing distance from rocky habitat had a positive total effect on mean VeDBA (24.3%), showing contrasting total effects despite both resulting in higher step lengths and lower direct effects on mean VeDBA (Fig. 5).

In all cases, higher mean temperatures decreased step lengths and mean VeDBA, while longer step lengths resulted in higher mean VeDBA (Fig. 5). Breeding season had a positive total effect of 0.282 on mean VeDBA, indicating that quolls used more energy in breeding season than in non-breeding season (Additional file 1: Figure S5).

## Discussion

Our study evaluated the habitat selection and energy use of the endangered northern quoll in an active mining landscape. As predicted, we found that at the broad scale, quolls preferred rugged, rocky habitat, and used spinifex grassland, riparian habitat, and disturbed mining habitats in proportion to their availability. At the fine scale, quolls used all habitats less than rocky habitat during breeding season, but used mining and riparian habitats at similar amounts during non-breeding season. Moving through mining habitat increased energy expenditure (mean VeDBA), suggesting that these areas may impose higher energetic costs than natural habitats. This supports existing concerns for quolls regarding the continued expansion of large-scale mining disturbance in the Pilbara and highlights the sub-lethal threats to species living in human-altered landscapes.

Northern quoll broad-scale movement ranges had higher proportions of, and were closer to, rocky habitat compared to the broader landscape—consistent with previous findings [50, 97, 133]. By contrast, quolls used mining habitats in proportion to their availability. This suggests that the quolls here did overlap their movement ranges with mining habitat. In the Pilbara, mining often targets rocky habitat given they are typically rich in mineral reserves [46], and it is likely that the mined area in this study was once rocky habitat used by northern quolls [50, 56]. It is therefore likely that quolls did not move into this landscape post-disturbance, but that they were already there before mining began. Living in or near to mining disturbance may inflate quoll movement ranges—with larger ranges observed here than in natural landscapes [133]—which could suggest that quolls are required to move more in mining landscapes [134].

During the breeding season, fine-scale integrated step-selection analysis suggested that quolls avoided mine pits and waste dumps, as well as other disturbed land, spinifex grassland, and riparian habitat, relative to rocky habitat. Mining habitats had lower perceived vegetation cover (i.e., NDVI) and topographic ruggedness than rocky habitat, more similar to spinifex grassland for these two variables [135–137]. Avoidance of spinifex grassland by quolls in natural landscapes has been previously documented [53, 97], and the avoidance of mining habitats in breeding season suggests a potential lack of resources like food (e.g., *Ficus* species—which only occur in rocky habitats) or dens during breeding. All quolls tracked in breeding season were male, and male northern quolls are known to travel long distances when breeding, seeking females and food [89, 133]—likely increasing their energy requirements during this time. Anthropogenic landscape disturbances, like mining, can reduce the abundance of potential prey species such as reptiles [30, 138, 139], mammals [140, 141], birds [142], invertebrates [143, 144], and vegetation [145], meaning that disturbed mining habitats may not satisfy the high resource requirements of quolls during breeding season [31].

The avoidance of mining habitats by male quolls in breeding season may also be driven by a reduced chance of breeding success. Female animal abundance is often lower in human-disturbed landscapes compared to males [146, 147]. Further, female northern quolls require specific denning and environmental conditions (e.g., deep, cool dens) to protect their young during breeding season [47], which mining landscapes may not satisfy. This is supported by the fact that we were only able to collar one female northern quoll here. In highly fragmented landscapes—like mining habitats—northern quolls are often less abundant [148], and vast areas of non-rocky habitat can reduce genetic connectivity in the species [69, 149, 150]. To persist in fragmented landscapes like this, it often requires increased long-distance dispersal [29], which may be exacerbated in mining landscapes where naturally-fragmented habitats are broken up further. Considering that male northern quolls typically perish after their first breeding season [116, 151], successful annual reproduction is vital for population success [152]. Reduced breeding opportunities in mining habitats could decrease the likelihood of males using this habitat and decrease the amount of viable breeding habitat in the landscape.

Another potential explanation for the observed avoidance of mining habitats in breeding season may be due to risk avoidance, or the landscape of fear [153, 154]. Like natural landscapes where quolls avoid predators that thrive in spinifex grassland [53, 155], mining habitats often present comparable or greater threats from feral cats and dingoes [43, 54, 55, 156]. Built infrastructure, such as roads and waste dumps, can attract predators and increase predation risk [101, 157, 158]. Animals often use habitats based on their perception of what is low and high risk, with areas of high predation risk often avoided [159]. Disturbances such as artificial light and noise pollution (such as that associated with machinery, spotlights, and ore processing within the mine) can also interfere with foraging and mating, and force unnecessary movements for animals [10, 160–162]. These risks coupled with reduced body condition in the breeding season may make mining habitats appear more dangerous [163, 164], deterring quolls during this period.

In contrast, in non-breeding season at the fine scale, quolls used mine pits and waste dumps, and other disturbed land in similar amounts to rocky habitat. This also applied to riparian habitat (e.g., densely vegetated drainage lines). During this time, northern quoll body condition was improved compared to breeding season and movement ranges were considerably smaller. Small movement ranges and healthy body condition often reflect the use of resource-rich habitats [165, 166]. However, for quolls, the lack of breeding-driven movement for males in non-breeding season likely leads to the need for fewer resources [89], potentially contributing to better body condition [6]. It is possible that quolls during this time obtain sufficient food resources from natural rocky and riparian habitats [similar to natural landscapes; 97]—but take advantage of limited resources in mining habitats due to lower energy demands in this season. Optimal foraging theory [3, 167]—which postulates that foraging animals seek to maximise energy intake in the minimum time needed to gain nourishment [168, 169]—would suggest that quolls would avoid mining habitats regardless of season, due to decreased food resources here. However, mining structures like waste rock piles have some evidence of short-term denning by quolls [47, 170], including by males in this study. Therefore, this increased use may be explained by a game-theoretical approach [171, 172], where animals might settle for a lower-quality habitat above a certain threshold in a fragmented landscape, to avoid moving towards known high-quality sites which may be lost to competitors or increase predation risk [173].

Long term population maintenance requires effective energy use. However, the increased use of mining habitats here comes with an energetic cost. Accelerometers revealed that northern quolls expended the most energy when primarily using mining habitats, likely driven by faster movement and high-cost behaviours such as bounding, jumping, and galloping [71]. This supports the suggestion that mine sites contain fewer resources or are riskier than favoured rocky habitat, where energy expenditure was lower—probably due to more resting or foraging behaviours there. Other small mammals show similar negative behavioural responses to anthropogenic habitats, by increasing speed [174] or tortuous movements [9]. Lower resources in mining habitats may force quolls to either traverse these areas quickly or use more energy to find food and shelter, increasing their overall food requirements [175]. For example, cougars living in anthropogenically-disturbed landscapes use more energy and are required to consume more deer annually to meet their energy requirements [31]. In non-breeding season, quolls used riparian habitat similarly to rocky habitat, which can allow efficient movement between rocky habitats [53, 69, 97]. The use of this habitat slightly increased energy expenditure, but not as much as mining habitats or spinifex grassland. This suggests that in non-breeding season, despite using habitats that were more energetically costly, quolls may be able take advantage of mining and riparian habitats due to lower energy requirements overall. However, in breeding season when efficient dispersal is vital and energy requirements are already high, rocky habitats are likely favoured given the higher chance of breeding and increased resource availability.

## Conclusion

Anthropogenic disturbance can significantly influence animal movement and energy expenditure [10], as evidenced by our study on northern quolls. Rocky habitat is the most important habitat for quolls, despite the presence of disturbance in the landscape. The replacement of favoured natural habitats such as this with energetically-costly mining habitats may exacerbate risks for quolls and negatively impact movement—more than if less-favoured habitats such as spinifex grassland were disturbed [176]. Conservation strategies such as establishing or retaining movement corridors [e.g., rocky and riparian habitats; 177–179], creating artificial refuges [180, 181], revegetation or restoration [182–184], and invasive predator control [185], could enhance disturbed habitats for animal movement in disturbed landscapes [50, 186]. However, restoring disturbed mining habitats can be difficult [187, 188], and offsetting habitat destruction is problematic, with few demonstrable successes [189–191]. So, to conserve northern quolls, the preservation and protection of favoured habitats like rocky and riparian habitats, should be prioritised in areas with active mining. More emphasis should also shift from focusing only on the impacts of habitat destruction before or after disturbance, to also managing potential negative impacts on animals during disturbance [192].

### Supplementary Information

**Additional file 1**. Supporting information and results.

## Data Availability

The datasets collected and analysed during the current study are available in the Figshare repository, 10.6084/m9.figshare.24145374.

## Abbreviations

AICc: Corrected Akaike Information Criterion
GLMM: Generalised Linear Mixed Model
GPS: Global Positioning System
HDOP: Horizontal Dilution of Precision
iSSF: Integrated Step Selection Function
KDE: Kernel Density Estimation
MCP: Minimum Convex Polygon
PSEM: Piecewise Structural Equation Modelling
RSS: Relative Selection Strength
TRI: Topographic Ruggedness Index
UD: Utilisation Distribution
VeDBA: Vectorial Sum of Dynamic Body Acceleration
VHF: Very High Frequency
