# Supplementary material for: Movement ecology of an endangered mesopredator in a mining landscape
Source: Mov Ecol. 2024 Jan 17;12:5. doi: 10.1186/s40462-023-00439-5 (PMC10795371; doi:10.1186/s40462-023-00439-5)
Supplement: Supplementary file 1 — Additional file 1. Supporting information and results. [file 40462_2023_439_MOESM1_ESM.pdf]

Movement ecology of an endangered mesopredator in a mining landscape

Supplementary information

Cowan, M. A.[1, 2], Dunlop, J. A.[1, 2], Gibson, L. A.[3], Moore, H. A.[2, 3], Setterfield, S.A.[2], Nimmo, D. G.[1]

[1]Gulbali Institute, School of Agricultural, Environmental and Veterinary Sciences, Charles Sturt University, 386 Elizabeth Mitchell Drive, Thurgoona, NSW 2640, Australia.

[2]School of Agriculture and Environment, The University of Western Australia, Crawley, WA 6009, Australia.

[3]Department of Biodiversity, Conservation and Attractions, 17 Dick Perry Avenue, Kensington, WA 6151, Australia.

Appendix 1: Methods used to create environmental covariate layers for habitat selection analyses.

To create the map of habitat classifications, we applied semi-supervised classification of a normalised difference vegetation index (NDVI) layer [1-3], derived from Sentinel-2 imagery [4]. The NDVI layer for the habitat map was captured at a scale of 10 m and we used a consistent layer (captured in October 2021) for both tracking periods. Woodie Woodie had no fires between tracking periods and habitat features remained relatively consistent between time periods. NDVI is calculated using the near infrared (NIR) and red (RED) bands:

$$NDVI = \frac{NIR - RED}{NIR + RED}$$

For Sentinel 2 data the NIR band is band 8 and the RED band is band 4.

We used the semi-automatic classification (SCP) plugin [5], in QGIS to classify habitat classifications from NDVI imagery. Classified habitats included spinifex grassland, riparian habitat (which was mostly associated with heavily vegetated creek lines), disturbed land, and water. To define all disturbed land accurately, we then overlaid a disturbance raster layer (converted from a vector) provided by *ConsMin* which reflected all mining disturbance in the landscape and was used for reporting at *Environmental Protection Authority (EPA)* standards. Finally, because rocky habitat is crucial for northern quolls in the Pilbara [6-8], we overlaid a northern quoll potential natural denning habitat raster layer (converted from a vector) which was digitised manually by *Western Wildlife* by outlining all visible rocky features from high-resolution aerial imagery. These features included rocky outcrops, gorges, and rocky mesas, as these are the areas which provide denning habitat for northern quolls in the Pilbara [9, 10]. This resulted in a habitat raster of the following habitat types: spinifex grassland, riparian habitat (dense vegetation associated with creek lines), water, rocky habitat, and mining disturbed land. During breeding season, quolls were often tracked to dens within rocky waste dumps and mine pits, therefore, we split mining disturbed land into two types: 1) mine pits and waste dumps, and 2) other disturbed land (e.g., roads, buildings, and large cleared areas). The final map was cross-examined with the corresponding satellite imagery to ensure the accuracy of habitat feature classification [11].

The topographic ruggedness index (TRI) is defined as the difference in elevation between a cell and the eight cells surrounding it [12]. To create a TRI map for our landscape, we sourced

a high-resolution radiometric terrain-corrected digital elevation model (12.5 m scale) [13], and used the 'Terrain Ruggedness Index' function in QGIS to calculate TRI for each cell [14].

To create maps representing distance to disturbance and distance to potential denning habitat, we used the same disturbance and habitat vector layers provided by ConsMin that we used to create the habitat classification map. We converted these vector layers to distance rasters using the "rasterize" function in the 'raster' package in R. Each resulting cell of the respective rasters (10 m scale) reflected the distance from disturbed land or potential natural denning habitat.

To determine differences in environmental characteristics among habitat classifications, we extracted the mean NDVI value and the median topographic ruggedness for each habitat. To define the area from which to sample from, we combined the *observed* and *available* movement ranges (values were compared separately for *observed* and *available* ranges, but were very similar so they were combined for simplicity), using QGIS [14]. We clipped all habitat types to the combine *observed* and *available* area and extracted the NDVI and topographic ruggedness values using the 'extract' function in the "raster" package in R [15].

Table S1: The parameters used during kernel density estimation for the measurement of northern quoll movement ranges. Parameters show the buffer size, grid output, and the UTM zone for each individual (ID) when using the ad hoc method ($h_{ad\ hoc}$), referred to as 'reference scaled' in the package "rhr" [16].

ID	X buffer (ha)	Y buffer (ha)	Grid output	UTM zone
33425	8857	4973	100 x 100	51S
33421	2835	9112	100 x 100	51S
33427	5565	7078	100 x 100	51S
33411	3862	4569	100 x 100	51S
33413	2889	1492	100 x 100	51S
33423	1819	1892	100 x 100	51S
33422	2083	1619	100 x 100	51S
33415	573	860	100 x 100	51S
33412	679	876	100 x 100	51S

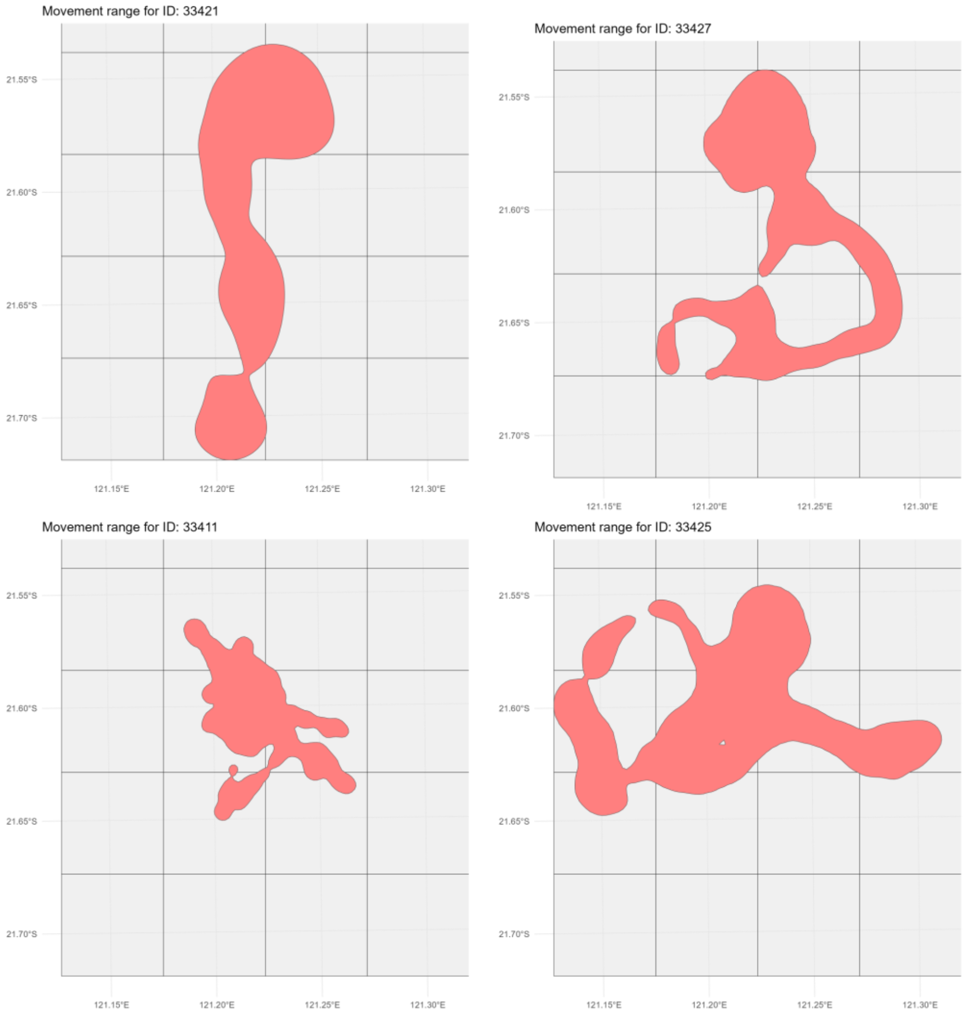

Figure S1: Northern quoll movement ranges during breeding season used in analysis. Individual ID is in the top left of each plot and each grid square is 5 km wide. Latitude is on the Y axis and Longitude is on the X axis.

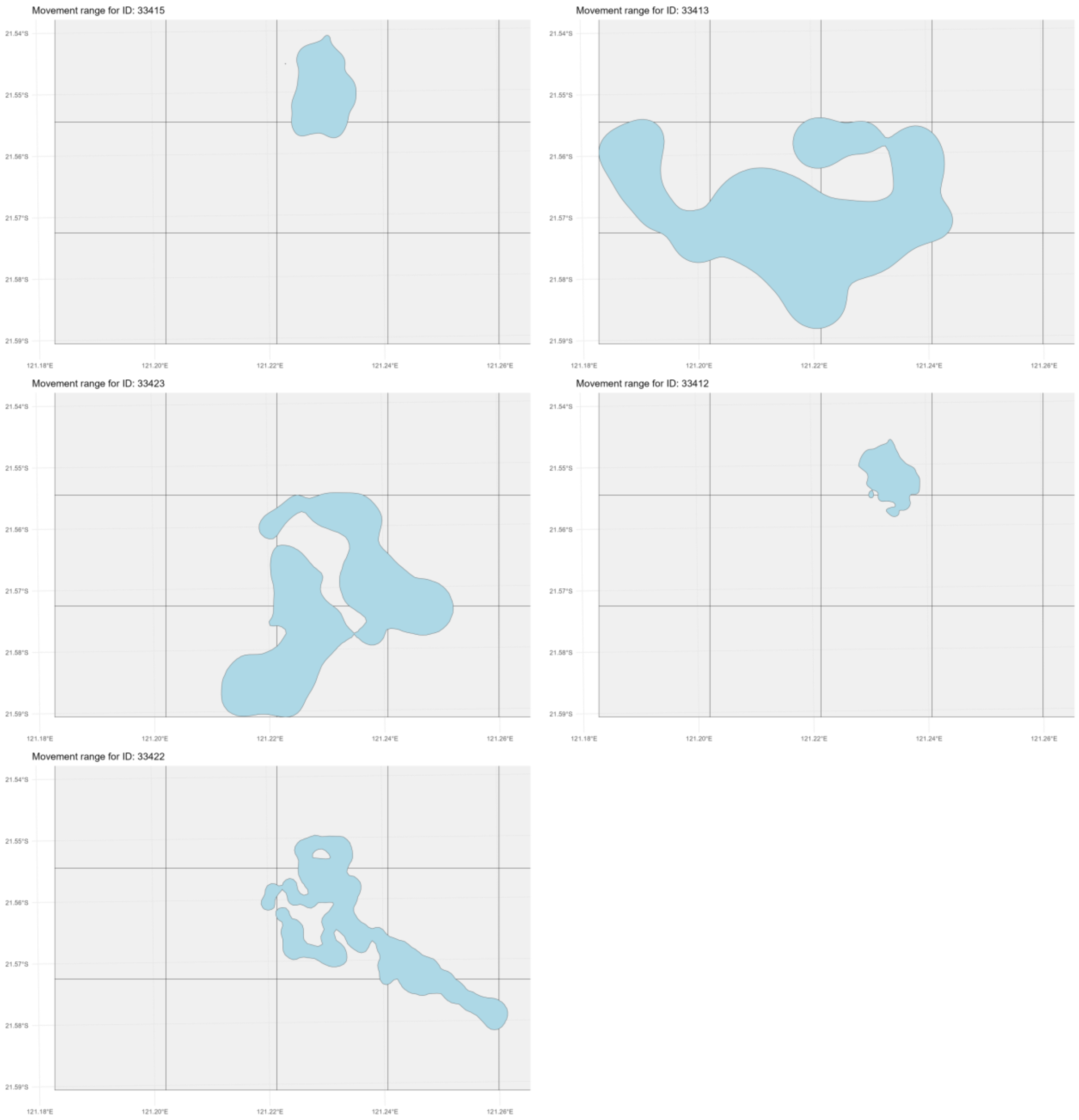

Figure S2: Northern quoll movement ranges during non-breeding season used in analysis. Individual ID is in the top left of each plot and each grid square is 2 km wide. Latitude is on the Y axis and Longitude is on the X axis.

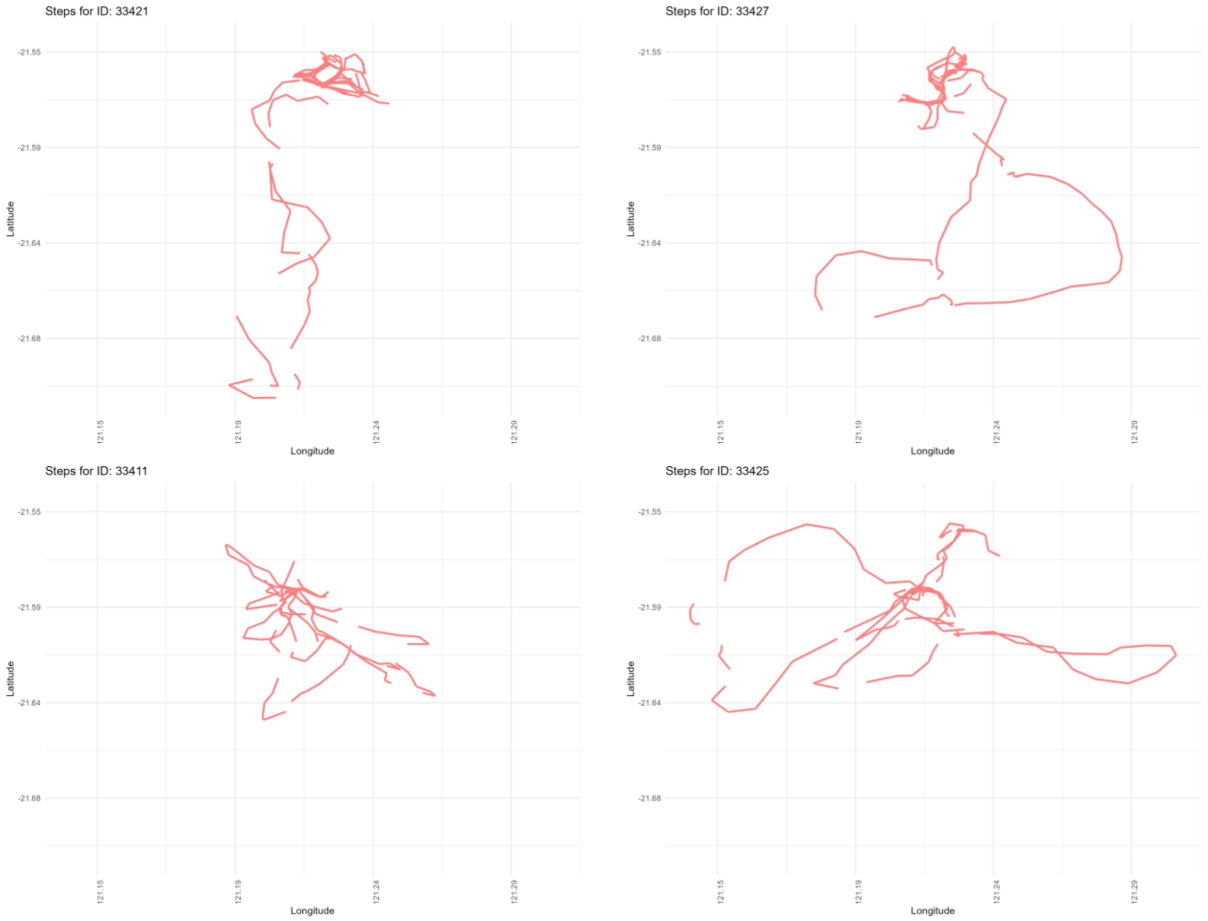

Figure S3: Northern quoll steps during breeding season used in analysis after data cleaning and removal of bursts with less than three steps. Individual ID is in the top left of each plot and each grid square is 5 km wide. Red lines signify steps.

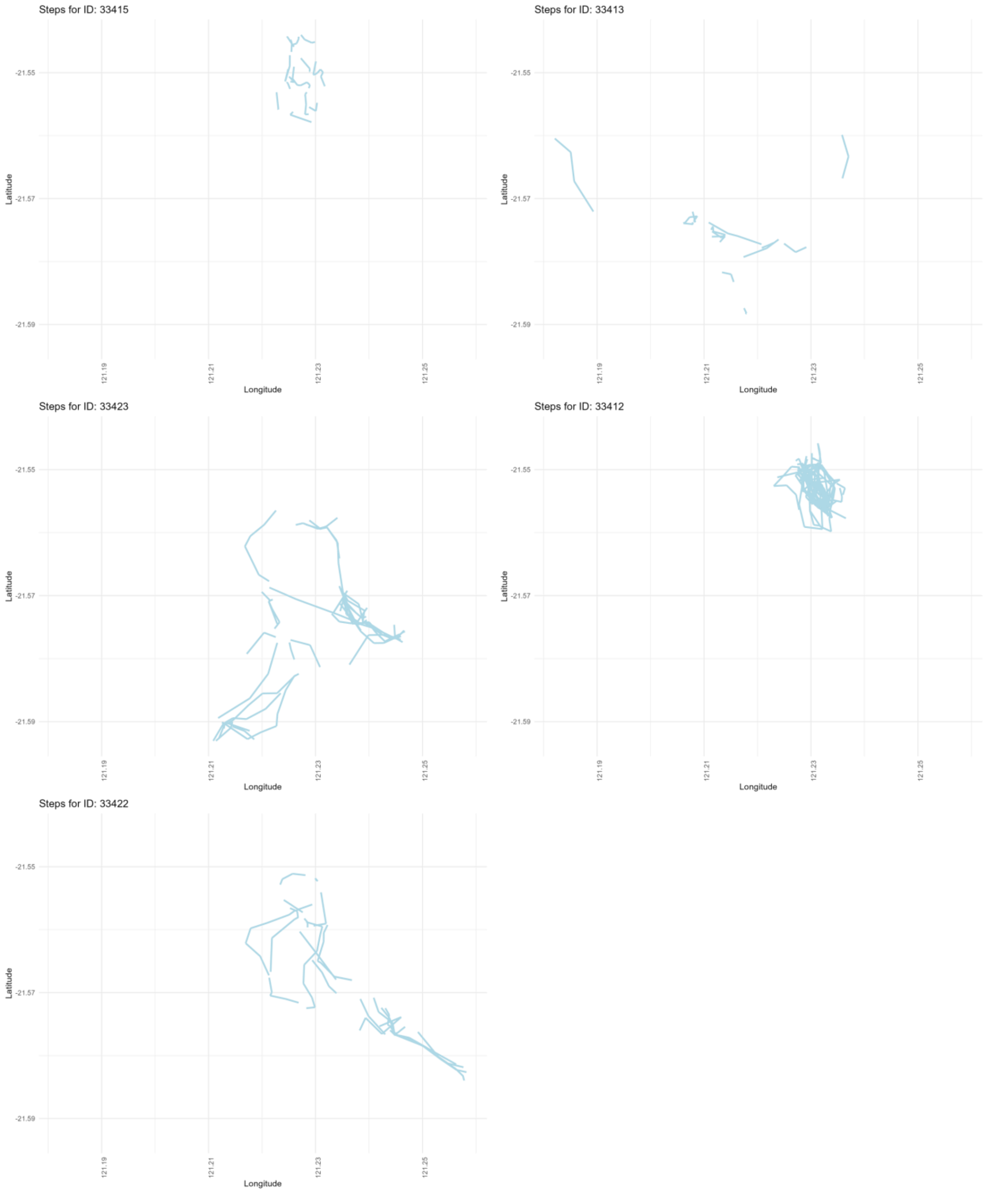

Figure S4: Northern quoll steps during non-breeding season used in analysis after data cleaning and removal of bursts with less than three steps. Individual ID is in the top left of each plot and each grid square is 2 km wide. Blue lines signify steps.

Table S2: iSSF models compared to determine the habitat selection of northern quolls in a mining landscape. Models compared include variables: habitat = habitat classification (spinifex sandplain, dense vegetation, rocky habitat, mine pits and waste dumps, other disturbed land), sl = step length, ta = turn angle, TRI = topographic ruggedness, HD = distance from potential denning habitat, DD = distance from disturbance, and age = age. The global model for both seasons is highlighted in bold.

Season	Mod. Num.	Model	K	AICc	Δ AICc	AICc Wt	Cum. Wt	LL
Breeding	3	habitat + log(sl) + cos(ta) + TRI + HD	8	5620.36	0	0.56	0.56	-2802.16
	**1**	**habitat + log(sl) + cos(ta) + TRI + DD + HD**	**9**	**5621.62**	**1.26**	**0.3**	**0.86**	**-2801.79**
	2	habitat + log(sl) + cos(ta) + TRI + DD	8	5624.21	3.85	0.08	0.94	-2804.09
	4	habitat + log(sl) + cos(ta) + TRI	7	5624.76	4.4	0.06	1	-2805.37
	6	habitat + log(sl) + cos(ta) + DD + HD	8	5633.94	13.58	0	1	-2808.96
	5	habitat + log(sl) + cos(ta)	6	5634.24	13.88	0	1	-2811.11
	8	TRI + log(sl) + cos(ta)	3	5682.96	62.6	0	1	-2838.48
	7	DD + HD + log(sl) + cos(ta)	4	5716.15	95.79	0	1	-2854.07
Non-breeding	4	habitat + log(sl) + cos(ta) + TRI + age × (log(sl) + cos(ta))	9	3960.35	0	0.26	0.26	-1971.15
	3	habitat + log(sl) + cos(ta) + TRI + HD + age × (log(sl) + cos(ta))	10	3960.69	0.33	0.22	0.48	-1970.31
	2	habitat + log(sl) + cos(ta) + TRI + DD + age × (log(sl) + cos(ta))	10	3961.16	0.81	0.17	0.65	-1970.55
	**1**	**habitat + log(sl) + cos(ta) + TRI + DD + HD + age × (log(sl) + cos(ta))**	**11**	**3961.51**	**1.16**	**0.14**	**0.79**	**-1969.72**
	5	habitat + log(sl) + cos(ta) + age × (log(sl) + cos(ta))	8	3961.78	1.43	0.13	0.92	-1972.87
	6	habitat + log(sl) + cos(ta) + DD + HD + age × (log(sl) + cos(ta))	10	3962.67	2.32	0.08	1	-1971.3
	7	HD + DD + log(sl) + cos(ta) + age × (log(sl) + cos(ta))	6	4038.75	78.4	0	1	-2013.36
	8	TRI + log(sl) + cos(ta) + age × (log(sl) + cos(ta))	5	4049.64	89.29	0	1	-2019.81

Table S3: Outputs of Bayesian zero-inflated regression models for broad scale use of each habitat type, and linear mixed-effects models for broad movement ranges and their median topographic ruggedness, mean distance from disturbance, and mean distance from potential denning habitat. Significant relationships are highlighted in bold and denote differences in used movement ranges from the intercept. The intercept is the *available* movement range.

Response Variable	Parameter	Estimate	Est.Error	L-95% CI	U-95% CI	Rhat	Bulk_ESS	Tail_ESS
Spinifex grassland cover	Intercept	1.48	0.18	1.12	1.85	1	1886	1649
	Observed	-0.47	0.32	-1.06	0.18	1	4214	2534
Dense vegetation cover	Intercept	-3.6	0.15	-3.89	-3.33	1	2319	2468
	Observed	0.09	0.26	-0.46	0.57	1	4906	2642
**Rocky habitat cover**	Intercept	-3.54	0.3	-4.15	-2.93	1.01	1217	1526
	**Observed**	**1.09**	**0.24**	**0.6**	**1.55**	**1**	**3308**	**2686**
Mine pits and waste dumps cover	Intercept	-2.08	0.21	-2.49	-1.69	1	2226	2005
	Observed	-0.31	0.37	-1.1	0.35	1	2588	2133
Other disturbed land cover	Intercept	-2.29	0.22	-2.75	-1.87	1	2126	1988
	Observed	-0.45	0.34	-1.18	0.18	1	3857	2543
**Median topographic ruggedness**	Intercept	0.62	0.04	0.53	0.71	1	1059	1152
	**Observed**	**0.18**	**0.05**	**0.08**	**0.27**	**1**	**4515**	**2482**
**Mean disturbance distance**	Intercept	1262.35	222.46	802.68	1687.8	1	2674	2300
	**Observed**	**-888.2**	**425.81**	**-1739.21**	**-53.87**	**1**	**4726**	**2838**
**Mean potential denning distance**	Intercept	1335.18	151.84	1035.73	1635.3	1	2494	1666
	**Observed**	**-826.47**	**290.45**	**-1397.02**	**-254.15**	**1**	**3856**	**2719**

Table S4: The outputs of iSSFs for models which had substantial support from AICc ranking (Table S3). Models are separated by their model number for each season. Response variables which were significantly different from the intercept (*p* = <0.05) are highlighted in bold. The intercept for categorical habitat classifications was rocky habitat.

Season	Mod. num.	Response variable	Coef.	Std. error	z-value	*p*-value	Lower 95% CI	Upper 95% CI
Breeding	3	**Spinifex grassland**	**-1.17**	**0.14**	**-8.47**	**<0.001**	**0.24**	**0.41**
		**Dense vegetation**	**-0.69**	**0.32**	**-2.12**	**0.034**	**0.27**	**0.95**
		**Mine pit and waste dump**	**-0.85**	**0.18**	**-4.65**	**<0.001**	**0.30**	**0.61**
		**Other disturbed land**	**-0.97**	**0.22**	**-4.47**	**<0.001**	**0.25**	**0.58**
		**log(step length)**	**0.09**	**0.05**	**2.07**	**0.038**	**1.01**	**1.20**
		cosine(turn angle)	0.02	0.08	0.29	0.773	0.87	1.21
		**Topographic ruggedness**	**0.31**	**0.08**	**3.84**	**0.000**	**1.16**	**1.59**
		**Distance from potential denning habitat**	**0.00**	**0.00**	**2.54**	**0.011**	**1.00**	**1.00**
	1	**Spinifex grassland**	**-1.17**	**0.14**	**-8.47**	**<0.001**	**0.24**	**0.41**
		**Dense vegetation**	**-0.71**	**0.33**	**-2.18**	**0.030**	**0.26**	**0.93**
		**Mine pit and waste dump**	**-0.81**	**0.19**	**-4.33**	**<0.001**	**0.31**	**0.64**
		**Other disturbed land**	**-0.93**	**0.22**	**-4.24**	**<0.001**	**0.26**	**0.61**
		**log(step length)**	**0.09**	**0.05**	**2.09**	**0.0370**	**1.01**	**1.20**
		cosine(turn angle)	0.02	0.08	0.30	0.7675	0.87	1.21
		**Topographic ruggedness**	**0.31**	**0.08**	**3.84**	**0.0001**	**1.16**	**1.59**
		Distance from disturbance	0.00	0.00	0.87	0.3844	1.00	1.00
		**Distance from potential denning habitat**	**0.00**	**0.00**	**2.15**	**0.0314**	**1.00**	**1.00**
Non-breeding	4	**Spinifex grassland**	**-1.17**	**0.12**	**-9.75**	**<0.001**	**0.25**	**0.39**
		**Dense vegetation**	**-0.68**	**0.32**	**-2.08**	**0.037**	**0.27**	**0.96**
		Mine pit and waste dump	-0.13	0.37	-0.34	0.732	0.43	1.82
		Other disturbed land	-0.80	0.43	-1.85	0.064	0.19	1.05
		log(step length)	0.11	0.07	1.57	0.116	0.97	1.28
		cosine(turn angle)	0.13	0.26	0.51	0.609	0.69	1.89
		Topographic ruggedness	0.14	0.08	1.86	0.062	0.99	1.34
		Age × log(step length)	-0.03	0.03	-0.95	0.342	0.92	1.03
		Age × cosine(turn angle)	-0.14	0.17	-0.79	0.433	0.62	1.22
	3	**Spinifex grassland**	**-1.10**	**0.13**	**-8.42**	**<0.001**	**0.26**	**0.43**
		Dense vegetation	-0.61	0.33	-1.84	0.066	0.29	1.04
		Mine pit and waste dump	0.06	0.40	0.16	0.874	0.49	2.33
		Other disturbed land	-0.67	0.44	-1.51	0.132	0.22	1.22
		log(step length)	0.12	0.07	1.66	0.096	0.98	1.29
		cosine(turn angle)	0.13	0.26	0.50	0.615	0.69	1.88
		Topographic ruggedness	0.13	0.08	1.64	0.101	0.98	1.32
		Distance from potential denning habitat	0.00	0.00	-1.25	0.210	1.00	1.00
		Age × log(step length)	-0.03	0.03	-0.89	0.375	0.92	1.03
		Age × cosine(turn angle)	-0.14	0.17	-0.79	0.429	0.62	1.22
	2	**Spinifex grassland**	**-1.17**	**0.12**	**-9.75**	**<0.001**	**0.25**	**0.39**
		**Dense vegetation**	**-0.68**	**0.32**	**-2.08**	**0.038**	**0.27**	**0.96**

	Mine pit and waste dump	-0.20	0.37	-0.52	0.601	0.39	1.71
	**Other disturbed land**	**-0.87**	**0.44**	**-1.99**	**0.047**	**0.18**	**0.99**
	log(step length)	0.10	0.07	1.37	0.172	0.96	1.27
	cosine(turn angle)	0.12	0.26	0.47	0.636	0.68	1.87
	**Topographic ruggedness**	**0.16**	**0.08**	**2.01**	**0.044**	**1.00**	**1.36**
	Distance from disturbance	0.00	0.00	-1.09	0.277	1.00	1.00
	Age × log(step length)	-0.02	0.03	-0.56	0.579	0.93	1.04
	Age × cosine(turn angle)	-0.13	0.17	-0.76	0.450	0.63	1.23
1	**Spinifex grassland**	**-1.10**	**0.13**	**-8.39**	**<0.001**	**0.26**	**0.43**
	Dense vegetation	-0.60	0.33	-1.83	0.067	0.29	1.04
	Mine pit and waste dump	-0.01	0.41	-0.01	0.989	0.45	2.20
	Other disturbed land	-0.73	0.45	-1.64	0.101	0.20	1.16
	log(step length)	0.10	0.07	1.45	0.147	0.96	1.28
	cosine(turn angle)	0.12	0.26	0.46	0.643	0.68	1.86
	Topographic ruggedness	0.14	0.08	1.79	0.074	0.99	1.34
	Distance from disturbance	0.00	0.00	-1.08	0.279	1.00	1.00
	Distance from potential denning habitat	0.00	0.00	-1.25	0.212	1.00	1.00
	Age × log(step length)	-0.01	0.03	-0.49	0.623	0.93	1.04
	Age × cosine(turn angle)	-0.13	0.17	-0.76	0.448	0.63	1.23
5	**Spinifex grassland**	**-1.22**	**0.12**	**-10.43**	**<0.001**	**0.23**	**0.37**
	**Dense vegetation**	**-0.76**	**0.32**	**-2.35**	**0.019**	**0.25**	**0.88**
	Mine pit and waste dump	-0.21	0.37	-0.59	0.558	0.39	1.65
	**Other disturbed land**	**-0.90**	**0.43**	**-2.09**	**0.036**	**0.18**	**0.94**
	log(step length)	0.10	0.07	1.40	0.161	0.96	1.26
	cosine(turn angle)	0.13	0.26	0.50	0.616	0.69	1.88
	Age × log(step length)	-0.02	0.03	-0.87	0.386	0.92	1.03
	Age × cosine(turn angle)	-0.13	0.17	-0.77	0.443	0.62	1.23

Table S5: The mean, standard deviation (SD), minimum (Min), and maximum (Max) Normalised Difference Vegetation Index (NDVI) values for each habitat classification in the *observed* and *available* landscapes. As well as the median, interquartile range (IQR), minimum, and maximum Topographic Ruggedness Index (TRI) in the *observed* and *available* landscapes.

**NDVI**		**Mean**	**SD**	**Min**	**Max**
	Spinifex grassland	0.16	0.04	0.03	0.64
	Dense vegetation	0.36	0.11	0.13	0.97
	Rocky habitat	0.19	0.05	0.07	0.62
	Mine pits & waste dumps	0.09	0.04	0.00	0.98
	Other disturbed land	0.11	0.04	0.00	0.65
**TRI**		**Median**	**IQR**	**Min**	**Max**
	Spinifex grassland	0.500	0.375	0.00	6.000
	Dense vegetation	0.375	0.375	0.00	5.500
	Rocky habitat	1.250	1.000	0.00	4.625
	Mine pits & Waste dumps	0.500	0.375	0.00	6.875
	Other disturbed land	0.500	0.375	0.00	5.875

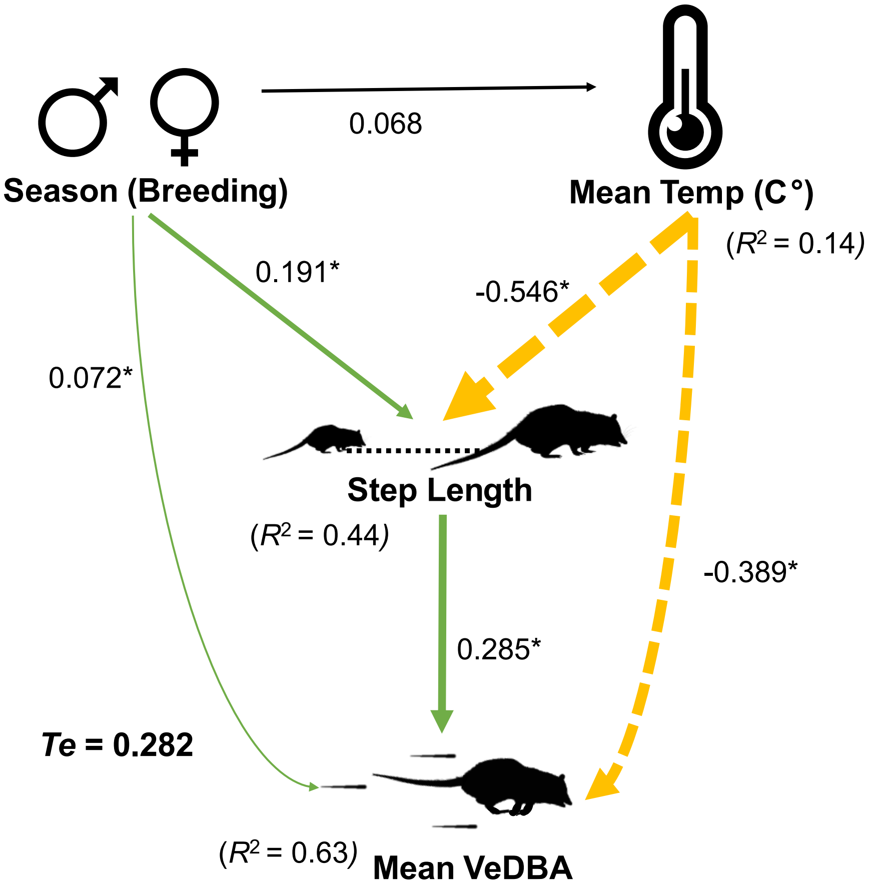

Figure S5: Relevant range coefficients for step length and mean VeDBA related to the influence of temperature and season (specifically, breeding season). Red dashed arrows represent a negative relationship and blue solid arrows represent a positive relationship. Arrow width shows the size of the effect, with wider arrows representing a larger effect. An asterisk signifies that the relationship is significant (p = <0.05) and the conditional R^2 value for step length and mean VeDBA is listed for each model, outlining the variance explained by the predictor variables. *Te* represents the total effect coefficient of season on mean VeDBA, both directly and mediated through step length. Icons adapted from Microsoft PowerPoint.

References

1. Pandey AC, Kulhari A. Semi-supervised spatiotemporal classification and trend analysis of satellite images. Bhatia SK, Mishra KK, Tiwari S, Singh VK, editors. Singapore: Springer Singapore; 2018. 353–63 p.

2. Cowan MA, Moore HA, Hradsky BA, Jolly CJ, Dunlop JA, Wysong ML, et al. Non-preferred habitat increases the activity area of the endangered northern quoll (*Dasyurus hallucatus*) in a semi-arid landscape. Australian Mammalogy. 2022;45(2):138–50.

3. Leroux L, Congedo L, Bellón B, Gaetano R, Bégué A. Land cover mapping using Sentinel-2 images and the semi-automatic classification plugin: A northern Burkina Faso case study. QGIS and Applications in Agriculture and Forest2018. p. 119-51.

4. USGS. Earth Explorer. 2023(Accessed 15 January 2023).

5. Congedo L. Semi-Automatic Classification Plugin Documentation. Release 6.0.1.1. 2016.

6. Moore HA, Michael DR, Dunlop JA, Valentine LE, Cowan MA, Nimmo DG. Habitat amount is less important than habitat configuration for a threatened marsupial predator in naturally fragmented landscapes. Landscape Ecology. 2022;37:935–49.

7. Moore HA, Michael DR, Ritchie EG, Dunlop JA, Valentine LE, Hobbs RJ, et al. A rocky heart in a spinifex sea: occurrence of an endangered marsupial predator is multiscale dependent in naturally fragmented landscapes. Landscape Ecology. 2021;36:1359–76.

8. Hernandez-Santin L, Goldizen AW, Fisher DO. Introduced predators and habitat structure influence range contraction of an endangered native predator, the northern quoll. Biological Conservation. 2016;203:160–7.

9. Hernandez-Santin L, Goldizen AW, Fisher DO. Northern quolls in the Pilbara persist in high-quality habitat, despite a decline trajectory consistent with range eclipse by feral cats. Conservation Science and Practice. 2022:e12733.

10. Cowan MA, Dunlop JA, Turner JM, Moore HA, Nimmo DG. Artificial refuges to combat habitat loss for an endangered marsupial predator: How do they measure up? Conservation Science and Practice. 2020;2(6):e204.

11. Tilahun A, Teferie B. Accuracy assessment of land use land cover classification using Google Earth. Am J Environ Prot. 2015;4(4):193–8.

12. Riley SJ, DeGloria SD, Elliot R. Index that quantifies topographic heterogeneity. intermountain Journal of sciences. 1999;5(1-4):23–7.

13. ASF DAAC. ALOS PALSAR_Radiometric_Terrain_Corrected_high_res; Includes Material © JAXA/METI 2009. In: DAAC A, editor. 2022.

14. QGIS Development Team. QGIS Geographic Information System. Open Source Geospatial Foundation Project. 2020.

15. R Core Team. R: A language and environment for statistical computing. R Foundation for Statistical Computing. 2022.

16. Signer J, Balkenhol N. Reproducible home ranges (rhr): A new, user-friendly R package for analyses of wildlife telemetry data. Wildlife Society Bulletin. 2015;39(2):358–63.
